# Mindfulness-based cognitive therapy and cognitive behavioral therapy for chronic pain in multiple sclerosis: a randomized controlled trial protocol

**DOI:** 10.1186/s13063-019-3761-1

**Published:** 2019-12-27

**Authors:** Dawn M. Ehde, Kevin N. Alschuler, Melissa A. Day, Marcia A. Ciol, Makena L. Kaylor, Jennifer K. Altman, Mark P. Jensen

**Affiliations:** 1 Department of Rehabilitation Medicine, School of Medicine, University of Washington, 325 9th Ave, Box 359612, Seattle, WA 98104 USA; 2 Department of Neurology, School of Medicine, University of Washington, UW Multiple Sclerosis Center, 1536 N 115th St, McMurray Building Suite 130, Seattle, WA 98133 USA; 3 School of Psychology, University of Queensland, 330 McElwain Building, Brisbane, Queensland 4072 Australia

**Keywords:** Multiple sclerosis, Chronic pain, Mindfulness, Mindfulness-based cognitive therapy, Cognitive behavioral therapy, Telehealth, Psychology

## Abstract

**Background:**

Chronic pain is one of the most prevalent and disabling symptoms associated with multiple sclerosis (MS). Individuals with MS are interested in nonpharmacologic pain management approaches. Cognitive-behavioral therapy (CBT) is efficacious in improving MS-related pain outcomes. Mindfulness-based cognitive therapy (MBCT) is a promising, alternative approach. Little is known about moderators of these treatments’ outcomes, however. This article describes the study protocol for the first randomized controlled trial comparing MBCT, CBT, and usual care and examining treatment effect moderators in individuals with chronic pain and MS.

**Methods:**

We will conduct a single-center, randomized, single blind, parallel-group trial comparing MBCT, CBT, and usual care in adults with MS and chronic pain. Both interventions will be delivered via eight group sessions using videoconferencing technology. Primary (average pain intensity) and secondary outcomes (including pain interference, depressive symptoms, fatigue, and sleep) will be assessed pre-treatment, mid-treatment, post-treatment, and at 6-month follow up. Potential treatment moderators will be assessed pre-treatment. We hypothesize that participants randomly assigned to MBCT or CBT will report significantly greater reductions in average pain intensity than participants assigned to usual care at post-treatment (primary study endpoint) and 6-month follow up. We also hypothesize that mindfulness, pain catastrophizing, and behavioral activation pre-treatment will moderate response to both active treatments, but not response to usual care.

**Discussion:**

Findings will provide important new information about the efficacy and moderators of two nonpharmacologic pain management approaches delivered using technology to overcome common barriers to treatment access. The knowledge gained may lead to better patient-treatment matching and, ultimately, better pain treatment outcomes in MS.

**Trial registration:**

ClinicalTrials.gov, NCT03782246. Registered on 20 December 2018.

## Background

Chronic pain is one of the most prevalent and disabling symptoms associated with multiple sclerosis (MS) [1, 2]. Psychosocial interventions - including cognitive behavioral therapy (CBT), hypnosis, and mindfulness - are increasingly recommended for reducing chronic pain and its impact on functioning and mood, and evidence supports their use in MS. [3–5] However, similar to pharmacologic approaches, as much as 50% of those who try these approaches may not achieve satisfactory pain improvement. This may be due to a mismatch between a specific treatment and a specific individual. For example, CBT, which focuses on reducing negative thoughts, may not benefit those who do not have problematic levels of maladaptive thinking (e.g., pain catastrophizing) prior to treatment [6].

Increased understanding of moderating baseline factors has significant potential to improve patient-treatment matching and outcomes. We recently proposed the Limit, Activate, and Enhance (LA&E) model [6] for understanding moderators of psychosocial pain treatments. The model theorizes that different interventions are designed to “limit” maladaptive coping (e.g., decrease catastrophizing or use of avoidance behavior), others to “activate” adaptive coping (e.g., increase approach behaviors or relaxation strategies), and others to “enhance” existing strengths (e.g., tap into mindfulness skills to build on their use for pain management). Some treatments may have two or all three of these goals. Use of this model could improve patient-treatment matching, and therefore outcomes, by determining a priori those factors that make an individual more or less suitable to one treatment versus another on the basis of their baseline profile.

Within the LA&E framework, we propose that treatments can be classified according to the extent to which they predominantly limit, activate, and/or enhance patient factors. For example, CBT approaches to pain typically focus on *limiting* maladaptive cognitions and *activating* appropriately paced behavior, with less emphasis on enhancing strengths. In recent years, increased attention has been placed on developing “enhance-centric” interventions that build and extend an individual’s existing strengths as a way to facilitate more effective coping. In particular, acceptance-based and mindfulness-based strategies provide an alternative to deficit-focused interventions by taking a strengths-based approach to target a shift in one’s relationship to experience (i.e., thoughts, pain), as opposed to changing the experience. In this context, mindfulness-based cognitive therapy (MBCT) is a multifaceted pain treatment, which integrates such an enhance-oriented approach with the incorporation of mindfulness techniques (e.g., meditation), as well as techniques that target cognitive (e.g., stress-pain connection exercises) and behavioral (e.g., scheduling nourishing daily activities) aspects of coping [7] In this way, with respect to the LA&E model, MBCT seeks to enhance mindfulness skills, limit maladaptive cognitions, and activate approach behaviors.

This article describes the rationale, aims, and protocol for the first randomized controlled trial (RCT) comparing MBCT and CBT to usual care for chronic pain. This RCT will identify not only the unique benefits conferred by these two treatments but also for whom each treatment is most suitable. The first aim is to determine the efficacy of group-based, videoconference-delivered MBCT and CBT for reducing pain intensity (the primary outcome) and secondary outcomes in adults with chronic pain and MS. Despite preliminary evidence in other populations experiencing pain [8–13], MBCT for pain has not been examined in MS nor compared to CBT. We also intend to increase our ability to more effectively match patients to treatments by examining moderators of MBCT (a limit-activate-enhance intervention) and CBT (a limit-activate intervention).

### Aims

This study will address two specific aims with corresponding hypotheses:

1. Aim 1: to determine the efficacy of group-based, videoconference-delivered MBCT and CBT interventions, relative to usual care, in reducing pain intensity (the primary outcome) in adults with chronic pain and MS;
  Hypothesis 1 (primary study hypothesis): participants randomly assigned to MBCT or CBT will report significantly greater reductions in average pain intensity (primary outcome) relative to participants assigned to usual care at post-treatment (primary endpoint).
2. Aim 2: to increase our ability to more effectively match patients to treatments by identifying pain treatment moderators. Although on average we expect similar outcomes in MBCT and CBT, we expect that there will be individual differences in who responds to each treatment. Specifically, we anticipate that pre-treatment levels of mindfulness, behavioral activation (activity), and pain catastrophizing will be associated with treatment response for the active treatment arms. Thus, to address Aim 2, we will explore pre-treatment levels of mindfulness, behavioral activation, and pain catastrophizing as predictors of response to MBCT and CBT;
  Hypothesis 2a: pre-treatment pain catastrophizing will be positively associated with treatment response for the two active treatment arms, but not the control condition;
  Hypothesis 2b: pre-treatment behavioral activation will be positively associated with treatment response for the two active treatment arms, but not the control condition;
  Hypothesis 2c: pre-treatment mindfulness will be positively associated with treatment response to MBCT but not to either CBT or the control condition.

In addition to testing these specific hypotheses, we will use study data to address the following exploratory aims:

1. The effects of MBCT and CBT relative to each other on both the primary (i.e., average pain intensity) and secondary outcomes (pain interference and key co-morbid symptoms including fatigue, sleep, and depressive symptoms), as hypothesis 1 pertains only to the effects of CBT and MBCT relative to the control, not to each other;
2. The relative effects of all three treatment conditions on the secondary outcomes;
3. The maintenance, loss or gain in any treatment effects at 6 months post-treatment;
4. Dose effects; and
5. Additional potential moderators of outcome, including demographics, baseline pain characteristics (e.g., pain severity, pain type) and baseline depressive symptom severity and fatigue.

## Methods/design

### Overview

**Fig. 1:**
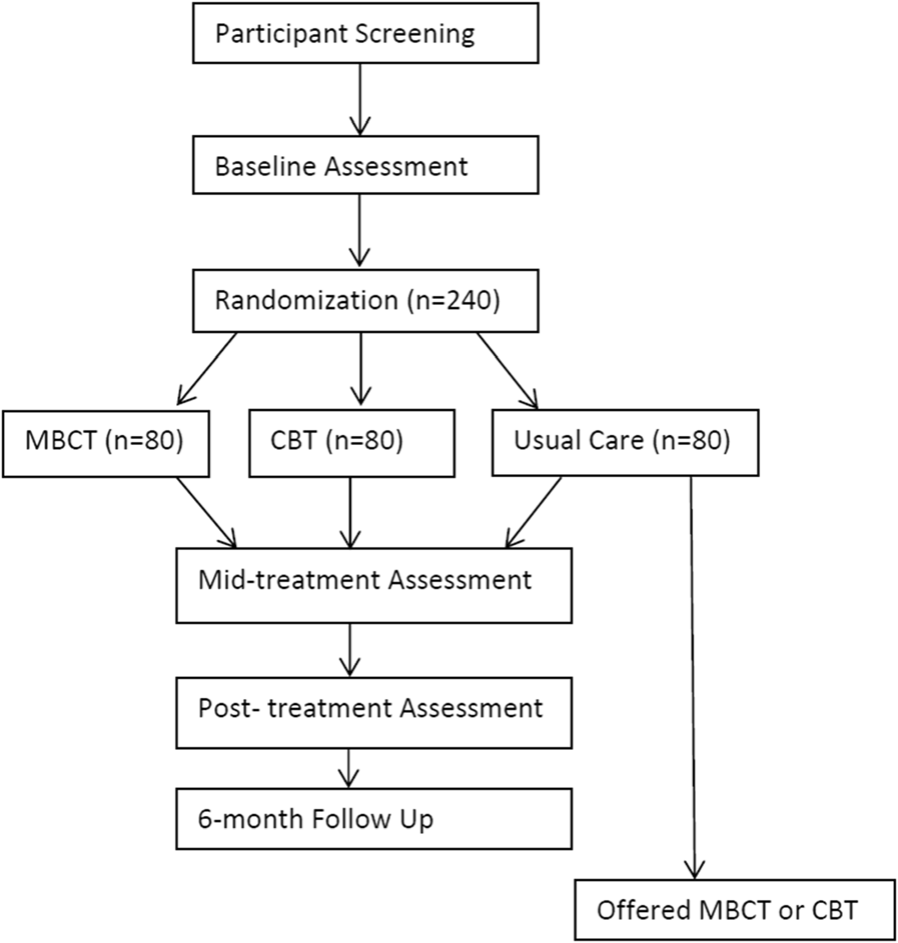
Study overview

This study is a three-group parallel (1:1:1), single-blind, randomized controlled trial comparing two-group-based, videoconference-delivered, nonpharmacologic pain treatments to usual care (see Fig. 1). Specifically, we will compare eight sessions of videoconference group delivery of MBCT and CBT to usual care for chronic pain in 240 adults with MS. Self-report measures of average pain intensity (primary outcome) and secondary outcomes will be assessed pre-treatment, mid-treatment, post-treatment, and at 6-month follow up. The MBCT and CBT intervention groups will start at the same time during week 1. Patients will be randomized immediately after the pre-treatment assessment.

We considered whether to compare MBCT and CBT only (two comparators) or MBCT, CBT, and a non-skill-building focused intervention such as a pain education group (three comparators). Most pain trials that compare active treatments, including pain education conditions, tend to find more similarities in outcomes, on average, than differences. Thus, adding an education control to this study would likely replicate those findings and not provide information as to whether engaging in these treatments yields additional benefits beyond what individuals are already doing in their day-to-day lives to manage pain (either effectively or ineffectively). Critically, this study will identify not only the unique benefits conferred by these two treatments relative to usual care - if significant differences emerge - but may also identify for whom each treatment is most suitable by examining moderators of treatment effects.

We will report the participant flow and study procedures following the Consolidated Standards of Reporting Trials (CONSORT) guidelines [14]. The trial was registered on ClinicalTrials.gov prior to enrollment of the first participant.

### Study setting

A sample of 240 participants (to achieve the goal of 204 completers; see power analysis below) will be recruited via a variety of regional and national sources. The study will be conducted out of a single site, the University of Washington (UW) Medicine MS Center in Seattle, WA, USA, although treatment will occur via telehealth and thus will be delivered across the USA and Canada. The trial will be supervised by the study investigators and trial manager, under the leadership of the principal investigator (DE). At weekly team meetings the investigators and the trial manager will meet with study staff to oversee and monitor study progress.

### Participant eligibility, recruitment, and consent procedures

Inclusion criteria are (1) 18 years of age or older; (2) a diagnosis of clinically definite MS confirmed by participant’s provider; (3) the presence of chronic pain, defined as average pain intensity in the past week of at least moderate severity (defined as ≥ 3 on the 0–10 numerical rating scale (NRS)) [15] and pain of at least 3 months duration, with pain reportedly present on at least half the days in the past 3 months; (4) reads and speaks English; (5) has access to and is able to communicate over the telephone; and (6) has a computer or digital device with video capabilities (any operating system) with Internet access. Exclusion criteria are (1) severe cognitive impairment defined as ≥ 2 errors on the 6-item Cognitive Screener [16]; (2) currently in psychotherapy or counseling for pain more than once a month; and (3) previously participated in a pain study that used CBT or MBCT.

Participants will be primarily recruited through advertisements on national websites, including the National MS Society’s website and ClinicalTrials.gov, as well as through national consumer publications (e.g., Momentum, the National MS Society’s magazine). At the UW MS Center, providers may directly refer patients to the study. Individuals may also self-refer to the study upon viewing a study brochure or flyer posted in the UW MS Center or in the community. If necessary to reach the required sample size, we will also recruit via targeted mailings to individuals in our UW MS research registry of > 1400 adults with MS from across the USA.

Individuals interested in the study will be invited to contact research staff by phone or email to schedule an initial eligibility screening. Staff will explain the study via telephone screening and confirm eligibility of potential participants. Staff will track the recruitment outcome for individuals who are deemed ineligible or who opt out of participating prior to completion of the consent process, including reasons for ineligibility or declining participation. Those who are eligible and remain interested in the study will engage in the informed consent process by phone. We will ask individuals who decline participation their reason for declining. Interested individuals will be asked to read the consent form and provide verbal consent. On the consent form, participants will be asked if they agree to use of their data should they choose to withdraw from the trial. Participants will also be asked for permission for the research team to share relevant data with people from the University of Washington taking part in the research or from regulatory authorities, where relevant. This trial does not involve collecting biological specimens for storage. Using Health Insurance Portability and Accountability Act (HIPAA)-compliant procedures, we will ensure potential participants meet the McDonald 2010 criteria for MS diagnosis by obtaining medical provider confirmation of clinically definite MS diagnosis, date of diagnosis, and MS disease course type.

### Study interventions and usual care condition

Both active interventions will consist of eight 2-h, group-based sessions delivered by postdoctoral psychology fellows, licensed psychologists, or a masters-level social worker (the “therapist”). Group delivery requires less therapist time (i.e., one therapist can treat 6–9 patients at once) and therefore may be more easily implemented in community settings in greater numbers of people with pain and MS. Group delivery also has the advantage of allowing participants to learn and obtain support from one another. The sessions will be offered at a variety of times (morning, afternoon, evening, weekend) to reduce barriers to participation and encourage adherence.

The interventions will be delivered in groups of 6–9 participants using the HIPAA-compliant Internet Zoom platform (https://zoom.us/). Zoom-hosted videoconferences allow all participants to see and hear one another. They also allow screen sharing, giving therapists the opportunity to display visual information (e.g., PowerPoint slides with session content) for all participants to view during the sessions. In the event that a participant cannot access the videoconference during any particular session, we will provide participants with a workbook (electronic and/or paper format) to follow during the sessions and facilitate skills practice outside of treatment sessions. Zoom also allows participants to call into the audioconference portion of the session in the event that they are unable to gain online access for any reason. Although both MBCT and CBT are compatible with either group or individual, face-to-face delivery, we adapted both for videoconference delivery via the Zoom platform (e.g., includes slides of in-session activities shared using screen-sharing).

Participants in any of the three conditions will not be encouraged or prohibited from using other pharmacologic or nonpharmacologic pain treatments. At each outcome assessment we will collect information about participants’ use of other concomitant treatments (pharmacologic and nonpharmacologic) to allow us to describe and compare treatment use across all three groups, including usual care. There will be no special criteria for discontinuing or modifying allocated interventions.

#### Mindfulness based cognitive therapy (MBCT)

We will use the 8-session MBCT for pain treatment manual and workbook [7] developed previously by one of the study investigators (Dr. Day) as the foundation of our MBCT intervention. This MBCT for pain protocol integrates cognitive and behavioral therapy techniques with mindfulness-based intervention strategies to form a streamlined approach to training the mind to respond more adaptively to pain. Participants in MBCT will be taught to apply the skills they learn not only to pain but also the problems pain causes for them including sleep disturbance, depressed mood, stress, and other problems. Each session will include at least one mindfulness meditation practiced in the group. Participants will also be given digital audio recordings of mindfulness exercises to facilitate practice of the mindfulness meditations prescribed as part of their homework.

#### Cognitive behavioral therapy (CBT)

We will use the CBT for pain treatment manual and workbook developed by Drs. Ehde and Jensen and tested in our prior RCT in MS [3, 17] as the foundation of the CBT intervention. The CBT intervention includes education about the role of cognitions (particularly pain catastrophizing), pain beliefs (including perceived control), and maladaptive or unhelpful coping behaviors in chronic pain; instruction in how to identify and change or restructure unhelpful or negative thinking about pain; utilization of positive coping strategies including positive coping self-statements; relaxation techniques; behavioral activation (including setting goals for activation), activity pacing and scheduling; and coping with pain flare-ups. Similar to MBCT, CBT participants will be taught to apply the skills they learn to both pain and its associated problems such as depressed mood, stress, and sleep disturbance. Each session will include a brief relaxation exercise practiced in the group. Participants will also be given digital audio recordings of the relaxation exercises to facilitate practice of the relaxation exercises prescribed as part of their homework.

#### Usual care

Participants randomly assigned to usual care will be contacted by telephone by a staff member not involved in data collection who will inform them of their allocation to usual care. Study personnel will not make any further attempts to influence usual care participants’ pain management or health care unless a psychiatric emergency arises (e.g., suicidal ideation is detected at an outcome assessment). Individuals assigned to usual care will be offered the opportunity to participate in their choice of either MBCT or CBT after they have completed their obligations for study data collection (i.e., after the 6-month follow up assessment). This will ensure that all study participants have the opportunity to receive an active treatment and increase retention of participants in the usual care control group.

### Therapist training, supervision, and fidelity monitoring

All study therapists will participate in a minimum of 14 h of initial training in the study population and interventions led by the study investigators, including Dr. Ehde (CBT expert), Dr. Day (MBCT expert), and Dr. Alschuler (MS population expert). They will also be trained in best practices for telehealth and group treatment delivery methods. Training will include readings, didactics, rehearsal of sessions, weekly supervision meetings, and coaching by the investigators, all of whom have substantial clinical expertise in the study population (people with MS and chronic pain), group delivery methods, and the study interventions.

The treatment fidelity protocol will include therapist manuals, protocol checklists (of prescribed, proscribed, common, and unique elements as well as adherence and quality ratings), weekly group consultation/supervision meetings with the supervising investigators (Drs. Ehde, Day, Jensen, and/or Alschuler), and an ongoing independent review of randomly selected digital recordings from 25% of all sessions. If fidelity problems or drift are detected at any point in the trial, the investigators will provide corrective feedback, additional coaching/practice, and ongoing monitoring until the therapist is delivering the treatment as intended. These procedures will ensure that the distinction between the conditions is maintained for the duration of the study.

### Videoconference technology orientation procedures

After the pre-treatment assessment, a staff member will conduct a training session for each participant to train them in use of the Zoom videoconferencing platform used to deliver the treatment sessions. Participants may use Zoom on whatever personal device with Internet access that they prefer, including desktop computers, laptops, tablets, or smartphones. Staff will send the participant an invitation to join a test meeting where the participant will be instructed in how to download Zoom. During this training they will give a brief overview on how to operate the basic Zoom functions that will be used in the treatment sessions, confirm that they can see the staff’s screen, and practice turning the audio and video on and off. This training will serve as an opportunity to address any technological issues, concerns, or questions the participant may have with their smartphone, computer, webcams, microphones, Zoom videoconferencing software, or with any other technology they will be using (e.g., a tablet). For those allocated to one of the two interventions, a staff member not involved in data collection will offer group refresher training one week before the sessions begin.

Several other procedures will be used to support participants’ use of the videoconferencing technology and to minimize technological disruptions during treatment. An unblinded staff member will log into the Zoom conference 30 min prior to the first treatment session, to be available to troubleshoot any technology issues that arise. Participants will be encouraged to sign in during this half hour to confirm that they are able to connect to the session and to receive assistance as needed. We will also provide participants allocated to MBCT or CBT with written instructions on using the videoconference platform and etiquette guidelines for participating in the videoconference calls.

### Randomization, allocation concealment, and procedures to minimize bias

Enrolled participants who complete the pre-treatment assessment will be randomly assigned to one of the three conditions. The trial manager is not involved in any outcome assessments and therefore will carry out randomization following the protocol designed by the study biostatistician (Dr. Ciol) prior to the study’s start. Participants will be randomized in stratified blocks using a computer-generated, password-protected randomization list, with randomization stratified by sex and pre-treatment pain intensity (mild/moderate or severe) to control for variation in outcome attributable to each stratification variable. The trial manager will notify participants of their allocation. All staff involved in data collection and management will be kept unaware of the participants’ group assignments and will explicitly inform participants that they (staff) are to remain blinded during the course of the study. The biostatistician will also be unaware of treatment assignment. Participants will be informed that the CBT and MBCT are each a type of pain self-management intervention and that the study’s purpose is to determine which treatment is more beneficial. Thus, only the staff involved in data collection and management are blinded, whereas study participants and the trial manager will have access to group allocation. We do not anticipate any requirement for unblinding during the course of the trial.

### Data collection procedures and measures

As depicted in Fig. 1, data will be collected pre-treatment, mid-treatment, post-treatment (primary endpoint), and at 6 months post-treatment. The primary outcome, secondary outcomes, descriptive, clinical, moderator, and process variables along with their associated time points of administration and supporting references are listed in Table 1. The study measures have evidenced reliability and validity for use in people with MS. Staff unaware of treatment allocation will collect the study measures via a combination of telephone interview and the Research Electronic Data Capture (REDCap) web-based data collection platform [39]. REDCap is a secure, HIPAA-compliant, password-protected, web-based data platform hosted by the University of Washington.

**Table 1:** Study measures and assessment timepoints

	Measures	Pre	Mid	Post	f/u
Descriptive and Clinical Variables					
Demographic characteristics	Age, sex,^a^ gender,^a^ race, ethnicity, relationship status, education (years), residence (country/state/zip code), employment status, all assessed by self-report	X			
Clinical variables	MS duration and diagnosis date^b^ MS severity: Expanded Disability Status Scale-self-report [18] MS course: NINDS CDE self-report questions on course [19]	X			
Pain characteristics	Location of pain sites PainDETECT: pain type, including neuropathic pain [20]	X			
Medications and treatments	List of all pain medications, disease modifying therapies, & non-pharmacologic pain treatments	X	X	X	X
Primary Outcome					
Pain intensity	The 0–10 numeric rating scale of average pain intensity in past week [21]	X	X	X	X
Secondary Outcomes					
Other pain intensity outcomes	Percentage with a meaningful improvement in average pain intensity (≥ 30% reduction from pre-treatment) Least, worst, and present pain assessed with 0–10 numeric rating scale	X	X	X	X
Pain interference	Brief Pain Inventory–Interference scale (modified version for MS) [21]	X	X	X	X
Physical function	PROMIS-29: 4 item version [22]	X	X	X	X
Depressive symptoms	Patient Health Questionnaire – 9 [23]	X	X	X	X
Fatigue severity	Modified Fatigue Impact Scale [24]	X	X	X	X
Sleep disturbance	PROMIS Sleep Disturbance scale [25]	X	X	X	X
Pain self-efficacy	UW CORR Pain Self-Efficacy Scale [26]	X	X	X	X
Treatment satisfaction	Patient Global Impression of Change [27] Patient Global Assessment of Treatment Satisfaction [27]			X	X
Global quality of life	Global Quality of Life Scale [28]	X	X	X	X
Primary Moderators					
Pain catastrophizing	Pain Catastrophizing Scale [29]	X	X	X	X
Mindfulness	Five Facet Mindfulness Questionnaire [30]	X	X	X	X
Behavioral activation	Behavioral Activation for Depression Scale [31]	X	X	X	X
Exploratory Moderators					
Pre-treatment pain intensity	The 0–10 numeric rating of average pain intensity [21]	X			
Positive affect	Positive Affect scale of the Positive and Negative Affect Scale [32]	X	X	X	X
Pain acceptance	Chronic Pain Acceptance Questionnaire [33]	X	X	X	X
Pain beliefs	Survey of Pain Attitudes: 2-item versions of harm and control subscales [34]	X	X	X	X
Pain resilience	Pain Resilience Scale [35]	X	X	X	X
Tx outcome expectancy	5-point Likert scale	X			
Cognitive functioning	Brief Test of Adult Cognition by Telephone [36]	X			
Age, sex, race/ethnicity	Demographic variables	X			
Process and Treatment-related Variables					
Therapeutic alliance	Working Alliance Inventory [37]		X	X	
Group climate	Group Climate Questionnaire-Engage scale [38]		X	X	
Treatment dose	Number, frequency, and duration of sessions attended		X	X	

*pre* pre-treatment, *mid* mid-treatment, *post* post-treatment, *f/u* 6-month follow up, MS multiple sclerosis, *NINDS CDE* National Institute of Neurological Disorders and Stroke Common Data Elements, *PROMIS* Patient-Reported Outcome Measurement System, *UW CORR* University of Washington Center on Outcomes Research in Rehabilitation

^a^ Ascertained by self-report

^b^ Confirmed by physician or nurse practitioner

All staff have been trained in best practices for data collection and management by our laboratory’s research coordinator, who also supervises the staff and monitors data quality.

We will implement multiple procedures for maximizing participant retention and outcome assessment completion. We will offer the treatment sessions and outcome assessments at different times of day to accommodate participants’ schedules. Participants will receive up to US$175 remuneration for completion of outcome assessments; they are not compensated for participation in treatment sessions, however. They will be encouraged to complete each outcome assessment, even if they stop attending treatment sessions or miss a previous outcome assessment. Data completion and quality will be monitored and tracked by the research coordinator, and the principal investigator (DME) will review recruitment and retention reports during weekly meetings and quickly implement changes if needed. We have successfully used these and other strategies in our past trials, with a retention rate of 90–98% across similar studies [3, 4, 40]. Further details on the trial’s retention and data management procedures may be found in the Manual of Procedures.

#### Primary outcome: average pain intensity

The primary outcome will be change from pre-treatment to post-treatment in average pain intensity over the past week assessed using an 11-point NRS, where 0 means “No pain” and 10 means “Pain as bad as you can imagine.” The NRS has consistently demonstrated its validity as a measure of pain intensity through its strong association with other measures of pain intensity as well as its sensitivity to detect changes in pain associated with pain treatment [27]. It has been validated in the MS population [21] and is considered a primary outcome measure for pain clinical trials per the recommendations of an expert consensus panel [27].

#### Secondary outcomes

Secondary outcome domains will include pain interference and the key co-morbid symptoms of fatigue, sleep, and depressive symptoms (see Table 1). Physical function, self-efficacy for managing pain, and quality of life will also be assessed at each time point.

#### Moderator domains

The primary moderator variables will be catastrophizing, mindfulness, and behavioral activation (Table 1). Exploratory moderators include demographic variables (sex, age), pain characteristics (pre-treatment pain intensity, pain type (neuropathic versus non-neuropathic pain)), baseline cognitive functioning, treatment expectancies, and baseline levels of the study outcomes and co-morbid symptoms (depressive symptoms, fatigue, and sleep disturbance).

#### Descriptive and process measures

As shown in Table 1, a range of demographic and clinical variables will be assessed pre-treatment to characterize the sample. These will include postal zip code as an indicator of rurality. Participants will complete measures of pain variables and MS disease characteristics in order to characterize their clinical status pre-treatment. Measures of MS disease were drawn from the National Institute of Neurologic Disorders and Stroke Common Data Elements for MS. [19] MS diagnosis, date of diagnosis, and course will be confirmed with participants’ providers. We will also obtain information about pain treatments (pharmacologic and nonpharmacologic) at each outcome assessment to allow us to describe treatment use in all three groups, including usual care.

The study therapists will complete an adherence log after each group session, which will include the number and duration (in minutes) of sessions attended by those assigned to MBCT or CBT. Participants allocated to one of the two active treatment groups will complete measures of therapeutic alliance (with the group therapist) and group climate (group engagement), as these may influence outcomes and thus be described and included in analyses.

### Safety protocols

We have a suicide safety protocol, should any participant exhibit indications of possible risk of self-harm during any phase of the study. All staff will be trained by the principal investigator (DME) to implement the protocol if a participant has a score ≥1 on the suicide item (item 9) of the Patient Health Questionnaire-9 or makes any reference to self-harm or suicidal thoughts at any time during the screening, outcome assessments, or treatment procedures. Upon identification of possible risk of self-harm, staff will use the suicide risk assessment protocol, which assesses the risk of self-harm and provides instructions for enacting procedures to assure patient safety based on their assessed risk. The safety protocol also includes procedures for the provision or arrangement of any clinical care needed during or after the trial to address suicide risk or any other distress arising from study participation. We will also track adverse events per UW Human Subjects Division procedures, which include monitoring for possible adverse events (solicited and spontaneously reported) and other unintended effects of the study interventions or study conduct, managing them, and reporting them to the principal investigator, Human Subjects Division, and study sponsor, as indicated. A data monitoring committee is not required by the study sponsor due to the minimal risk of this trial’s study interventions and procedures.

### Statistical power

The primary study outcome variable will be change in average pain intensity, as represented by the difference between the pre-treatment and post-treatment average pain intensity measures. Anticipated effects for CBT and usual care are based on the changes observed in our previous CBT trials using the 0–10 NRS. Anticipated effect sizes for MBCT are based on published studies of the effects of treatments involving mindfulness in which 0–10 NRS measures were used. Assuming a decrease in pain score of 0.3 points in the usual care group, 0.8, 1.0, and 1.4 in the CBT group, and 0.6, 0.8, and 1.0 in the MBCT group, we calculated the sample size to find differences between pre-treatment and post-treatment differences in scores, with alpha of 0.05, power of 0.80, and varying the standard deviation (SD) from 0.15 to 1 (to cover values observed). Sample sizes of 68 completers per condition will provide at least 80% power, even at the largest standard deviation, to detect a significant between-group effect for pre-treatment to post-treatment changes in average pain intensity.

### Statistical analyses

We will report the number of participants approached (and recruitment source), eligible, excluded, declined, enrolled, randomized, and who provided data at each assessment point (including reasons for exclusion, declination, etc.). We will examine distributions of all variables for outlying values and skewness; where indicated, variables will be recoded or transformed. We will use descriptive statistics to summarize all data, including demographic, clinical, primary, secondary, and moderator variables. We will summarize intervention information, including session adherence, duration, and homework completion, as well as fidelity indicators.

Next, we will identify patterns in missing data. If there are very few missing data, we will analyze the complete dataset. If there is a substantial number of missing values in the outcomes (say, about 5% or more), we will need to decide if the data are missing completely at random (MCAR), i.e., missing data are independent of observable variable and unobservable parameters, and occur at random, or missing at random (MAR), i.e., missing values do not occur at random, but can be modeled by variables that have complete information, in which cases the analysis of the complete dataset still provides unbiased estimators. Both MCAR and MAR cannot be verified statistically, and assumptions need to be made. For example, if missing data for pain post-treatment were missing because those individuals had higher levels of pain, the missing pattern would not follow MCAR or MAR, and therefore would be non-ignorable. If this situation happens, we will implement multiple imputation procedures, where several imputed datasets will be created and analyzed, and their results combined. We propose 50 multiple imputations (the literature suggests 20–100) [41]. However, we will implement various checks in the protocol in order to minimize the number of missing values. We do not plan to conduct interim analyses and therefore do not have any stopping guidelines.

#### Testing hypothesis 1

We will use an intent-to-treat approach to test hypothesis 1: all randomized participants will be included based upon their assigned treatment group regardless of actual treatment received and adherence. Hypothesis 1 states that participants randomly assigned to MBCT or CBT will report significantly greater reductions in average pain intensity relative to participants assigned to usual care, post-treatment (primary endpoint). This will be tested using analysis of covariance, with treatment condition (MBCT, CBT, usual care) as the explanatory variable, the pre-treatment average pain intensity score as the covariate, and change from pre-treatment to post-treatment score in average pain intensity as the response variable. Support for hypothesis 1 would emerge if after controlling for the pre-treatment pain score, there are differences in the response variable by intervention group, and subsequent post-hoc analyses comparing the adjusted means indicate greater reductions in pain intensity in the MBCT and CBT conditions, relative to the usual care condition. Although we anticipate that MBCT and CBT will have similar effects on the primary outcome variable, these analyses will also allow us to compare, as an exploratory test, the relative effects of MBCT and CBT. Effect sizes will be computed and reported. Secondary outcomes will be assessed using similar analyses to those used to test hypothesis 1.

#### Testing hypotheses 2a–2c

To test the moderation hypotheses, we will use linear regression analysis. Change in pain intensity will be the response (criterion) variable. Pre-treatment measures of pain catastrophizing, mindfulness, and behavioral activation will be entered in the model as possible moderators; terms representing the interaction between treatment condition and the moderators will test hypothesis 2a–2c. If the coefficient for a certain interaction is statistically different from zero, this will be interpreted as a moderation effect of the moderator present in the interaction.

### Dissemination policy

The study results will be disseminated through several channels, including scientific presentations at national and international conferences and peer-reviewed scientific journal articles. Study results will also be posted on ClinicalTrials.gov under identifier NCT03782246. We will send all study participants an electronic newsletter summarizing the study findings and implications, and a link to the posted results on ClinicalTrials.gov. Results will also be posted to our laboratory website and through our UW social media accounts. The National MS Society will disseminate our study findings to MS providers, scientists, and the public through their website, newsletters, research updates, and social media accounts. Investigators will adhere to the International Committee of Medical Journal Editors guidelines for determining authorship of all presentations and publications. We do not intend to use professional writers.

## Discussion

This study was designed to address several critical gaps in the management of chronic pain. It is the first RCT to evaluate the efficacy of MBCT relative to CBT and usual care for chronic pain, which has not been done in MS nor in any other chronic pain condition. MBCT is a promising, innovative treatment that may benefit those individuals with MS who do not respond to CBT. MBCT integrates mindfulness meditation practices within a CBT-oriented framework to address not only unhelpful cognitions and behaviors but also other components central to effective pain management, such as attentional control, decoupling of attention from emotion, mindful cognitions, and meditative behavior, all of which are hypothesized to be influenced by MBCT [42]. Study findings will provide critical information about the relative benefits of both MBCT and CBT compared to one another and to usual care. The findings will also determine the value of both of these approaches as adjunctive pain interventions, and if results support the use of MBCT, will expand the currently available treatment options for people with MS and chronic pain.

In addition to evaluating the efficacy of MBCT and CBT relative to one another and to usual care, this study will address a key gap in our understanding of variability in treatment responses to two psychosocial pain interventions. Comparisons of pain psychosocial interventions often yield equivalent efficacy; however, within-group comparisons indicate that there are both responders and non-responders to specific treatments [43, 44]. That is, for any individual, two different treatments such as CBT and MBCT may not necessarily be similarly efficacious. There is an urgent need to understand those factors underlying this individual-level variability in responses across different psychosocial treatment interventions. This understanding will inform for whom different psychosocial pain interventions work. Such knowledge will lead to more precisely targeted patient-treatment matching and, ultimately, better treatment outcomes for chronic pain in MS. The present study aims to address this gap by examining pain treatment moderators of both CBT and MBCT. Moreover, this is one of the first studies to test the moderators of psychosocial treatments on the basis of an a priori theoretical framework - the LA&E model. This is important as Kazdin [45, 46] has described how the lack of theory guiding prior tests of moderation is likely a critical reason underlying why the psychotherapy field has failed to advance the development of patient-treatment-matching algorithms.

Psychosocial interventions are underutilized for addressing chronic pain, both in the general population and in people with MS, with one driver of this underutilization being limited access to such treatments [43, 47, 48]. People with chronic pain co-occurring with MS are particularly likely to have poor access to nonpharmacologic pain care [49]. To overcome this barrier, the proposed study capitalizes on an emerging delivery innovation in the form of web-based, group-delivered videoconferencing technology. The current standard delivery format for psychosocial pain interventions is in person, limiting access to only a subset of clinical settings and patients, typically those residing in urban locations. Leveraging available technology and delivering interventions in formats with broader reach affords the capacity to transcend geographical barriers and target larger, more diverse populations. Approaches such as videoconferencing also have inherent scalability potential, as it is easier to centralize and scale up such technologies for public health dissemination [50]. Remote delivery may also reduce stigma and lower the threshold for initiation of treatment, given that such technologies can be used in the privacy of one’s home and outside the mental health system. They also tend to be less expensive than traditional psychotherapies, thereby possibly making participation in such programs more viable for individuals from lower socio-economic backgrounds [50]. Although videoconferencing has increasingly been used to deliver a wide variety of healthcare interventions, rigorous RCTs of group-based videoconferencing are surprisingly absent from the literature on chronic pain. Thus, the results of this study will have important implications for overcoming access barriers and for treatment disparities in not only people with MS and pain, but also other populations experiencing pain.

As with any research design, this present study has a few potential limitations. The trial will be conducted out of a single study site, although people with a physician-verified diagnosis of MS who live anywhere in the USA will be eligible to enroll. The study sample will also be limited to individuals who have sufficient Internet access capabilities to use videoconferencing. Fortunately, Internet access and use is increasing rapidly among adults, with 92% of urban, 90% of suburban, and 78% of rural adults in the USA reporting use of the Internet in a 2018 national survey [51]. We will track and report the number of individuals interested in the study who do not have Internet access but are otherwise eligible to learn more about this limitation. This is the first clinical trial to evaluate group-based, videoconference delivery of MBCT and CBT. As such, there are no best practices or treatment guidelines to inform how to best deliver these treatments, including the experiential components of mindfulness meditation (in MBCT) and relaxation exercises (in CBT) over videoconference. Thus, there may be unanticipated challenges or problems that arise in the delivery of these treatments via videoconferencing. We will track such challenges, if they arise, as well as any technology problems that occur during the course of the study, to inform future implementation of videoconference group delivery.

In conclusion, this study seeks to expand the treatment options available to individuals with chronic pain and MS. As a group, people with MS are eager to learn nonpharmacologic strategies for managing symptoms such as pain [52]. Showing that MBCT is effective and that MBCT and CBT might be effective in different individuals has significant importance for clinical translation: this will allow for informed, a priori decisions about which treatment approach to deliver to which individual to efficiently and optimally obtain meaningful benefit for that individual*.* The study findings may also have relevance to other people experiencing chronic pain, including other people with neurologic conditions such as traumatic brain injury, where pain is common and access to evidence-based nonpharmacological treatment is limited.

## Trial status

The UW Human Subjects Division approved the study protocol (version 1) on 8 May 2018, and the first participant was enrolled on 27 December 2018. The treatment phase is anticipated to end in September 2021, with the final outcome assessments planned for May 2022.

## Data Availability

Data sharing is not applicable to this article as no datasets were generated or analyzed during the current study. The study investigators (DME, KNA, MAD, MAC, MPJ) will have access to the final trial dataset. A de-identified dataset used in the trial’s analyses will be available from the corresponding author on reasonable request. We will also select a data repository for eventual data sharing after completion of the trial and initial publications.

## Abbreviations

CBT: Cognitive behavioral therapy
LA&E: Limit, activate, and enhance model
MBCT: Mindfulness-based cognitive therapy
MS: Multiple sclerosis
NRS: Numeric Rating Scale
RCT: Randomized control trial
REDCap: Research Electronic Data Capture
UW: University of Washington
